# Mental health risk factors during the first wave of the COVID-19 pandemic

**DOI:** 10.1192/bjo.2021.1031

**Published:** 2021-10-27

**Authors:** Henrique Prata Ribeiro, André Ponte, Miguel Raimundo, Tiago Reis Marques

**Affiliations:** Faculty of Medicine, University Clinic of Psychiatry and Medical Psychology, University of Lisbon, Portugal; Psychiatry Department, Hospital do Divino Espírito Santo de Ponta Delgada, Portugal; Oftalmology Service, Centro Hospitalar e Universitário de Coimbra, Portugal; and Faculty of Medicine, University of Coimbra, Portugal; Psychiatric Imaging Group, MRC London Institute of Medical Sciences, Imperial College London, UK; and Department of Psychosis Studies, Institute of Psychiatry, Psychology and Neuroscience, King's College London, UK

**Keywords:** COVID-19, physical distancing, mental health, depression, anxiety

## Abstract

**Background:**

During the first wave of the COVID-19 pandemic, distancing measures were enforced to reduce virus spread, which likely had an impact on the overall mental health of the population.

**Aims:**

To investigate the prevalence of mental health outcomes (depression, anxiety and insomnia), and associated risk factors, during a physical distancing period imposed in the first wave of COVID-19.

**Method:**

During the first month of Portugal's state of emergency, an online survey was created and disseminated through social media channels. Sociodemographic and clinical variables were assessed via self-reported questionnaires. Univariate linear regressions were used to identify associations between the collected variables and mental health outcomes. Multivariate regression analyses were performed to identify independent risk factors for clinical outcomes, with adjustment for potential confounders.

**Results:**

We analysed data from 1626 participants: a significant proportion showed depression (30.2%), anxiety (53.1%) and insomnia (36.3%) symptoms. Multivariate regression models showed that being male and working from home were protective for all mental health outcomes analysed, whereas the perception of infection, being under psychiatric care and taking medication were risk factors (*P* < 0.05). Days in isolation and being unemployed were risk factors for depression and insomnia (*P* < 0.05). Younger age and being a student were risk factors for depression, whereas being a healthcare professional was protective (*P* < 0.05). Indirect contact with COVID-19 was a risk factor for anxiety (*P* < 0.05).

**Conclusions:**

COVID-19-related distancing measures were associated with high levels of adverse mental health symptoms. Several risk factors were associated with these symptoms, which highlight the importance of identifying vulnerable groups during physical distancing periods.

## Study rationale

During the first wave of the COVID-19 pandemic, many countries introduced aggressive physical distancing strategies, such as closing schools and businesses, cancelling sporting events and asking people to isolate themselves at home or in a dedicated quarantine facility, as a way to slow the spread of the outbreak.^1^

Preclinical and clinical studies have shown that social isolation induces widespread brain changes^2^ and is associated with several psychiatric symptoms and disorders.^3^ Studies in rodent and macaque showed that isolation promotes neurophysiological effects comparable to those seen in human mood disorders, such as increased activation of the hypothalamic-pituitary-adrenocortical axis and decreased brain-derived neurotrophic factor expression, associated with negative affective changes (anhedonia, anxiety, guilt, fear, aggression).^4–6^ Findings in human populations during prolonged periods of isolation, as observed in polar expeditions or solitary confinement in prisons, show that living in these conditions has an associated psychological toll, such as disturbed sleep, impaired cognitive ability and negative affect.^7,8^

## Past and present comparisons

Experience from recent epidemics (2002–2004 severe acute respiratory syndrome and 2015 Middle East respiratory syndrome outbreaks) shows that imposed distancing measures were accompanied by increased symptoms of depression, anxiety and post-traumatic stress disorder.^9–12^ As for the COVID-19 pandemic, researchers expect the impact on mental health to be wide-ranging, substantial and possibly long-lasting.^13^ Shi et al,^14^ in a Chinese population-based study, described a high prevalence of mental health symptoms, with rates of 27.9% for depression, 31.6% for anxiety and 29.2% for insomnia. In another study, conducted in China during the early stages of the COVID-19 pandemic, Wang et al^15^ reported that female gender, being a student and specific physical symptoms (e.g. myalgia, dizziness, coryza) were associated with a greater psychological effect of the outbreak and higher levels of stress, anxiety and depression. Specific up-to-date and accurate health information and certain precautionary measures were associated with a lower psychological effect of the outbreak and lower levels of stress, anxiety and depression.^15^ The present study was conducted during the initial stage of the COVID-19 pandemic in Portugal. Pre-pandemic studies show that in the Portuguese population there is an estimated prevalence of 9.32% for depression, 6.06% for anxiety disorders^16^ and 27.7% for insomnia symptoms;^17^ however, it is still unclear how the COVID-19 pandemic has affected the mental health status of the Portuguese population immediately following the lockdown in the first wave.

In this study, we used an online survey to assess depression, anxiety and insomnia symptoms in the general adult population during the first month of COVID-19 physical distancing. Moreover, we evaluated the potential associated risk and protective factors for these mental health outcomes.

## Method

### Study design

This is a cross-sectional study performed via an online survey disseminated through social media channels during a period of 1 month. Data was collected from the first day of the declaration of a state of emergency in Portugal (18 March 2020) to 18 April 2020. Only participants residing in Portugal, aged ≥18 years, who provided informed consent and completed the survey were included in the study. Electronic informed consent was obtained from all the participants. The study was validated by the Centro Hospitalar Psiquiátrico de Lisboa's Ethical Committee (approval number: 006/2020) and every participant gave their explicit consent for analysis and anonymised publication of the data.

### Measurements

The survey included questions on sociodemographic characteristics, such as gender, age and area of professional activity, as well as questions related to the characteristics of the participant's isolation: days in isolation, number of people in the participant's household, contact with COVID-19 and work arrangement at the time. Participants were also asked about their background history of mental health disorders, potential psychiatric care and previous psychiatric medication. Additionally, depressive and anxiety symptoms, as well as insomnia, were assessed, using three gold-standard self-reported questionnaires. Depressive symptoms were assessed with the Beck Depression Inventory (BDI), a 21-item questionnaire designed to evaluate the intensity of depressive symptoms in the past week, using a four-point Likert scale.^18^ The total score ranges from 0 to 63, with the highest scores indicating more severe depressive symptoms. Anxiety symptoms were assessed with the Beck Anxiety Inventory (BAI), a 21-item questionnaire with the aim of evaluating the intensity of anxiety symptoms in the past week, using a four-point Likert scale.^19^ The total score ranges from 0 to 63, with the highest scores indicating more severe anxiety symptoms. Insomnia was assessed with the Insomnia Severity Index (ISI), a seven-item self-reported questionnaire developed to measure the nature, severity and impact of insomnia in the past 2 weeks, using a five-point Likert scale.^20^ The total scores of these assessment tools are interpreted as follows, based on the established literature: BDI, normal (0–9), mild (10–18), moderate (19–29) and severe (30–63) depression; BAI, normal (0–7), mild (8–15), moderate (16–25) and severe (26–63) anxiety; and ISI, normal (0–7), subthreshold (8–14), moderate (15–21) and severe (22–28) insomnia. We used the Portuguese versions of the BDI,^21^ BAI^22^ and ISI,^23^ which are validated for the Portuguese population and show good psychometric properties.

### Statistical analysis

Collected data are described as means and proportions. Regarding inferential statistics, we used univariate linear regressions to identify associations between the collected variables and the clinical scores of depression, anxiety and insomnia (considered as linear outcomes). Variables with *P* < 0.10 were considered for analysis in the multivariate linear regression models. All hypotheses were tested at a two-sided significance level of *P* < 0.05. Analyses were conducted with Stata version 14 for Windows (StataCorp LLC, USA).

## Results

**Fig. 1:**
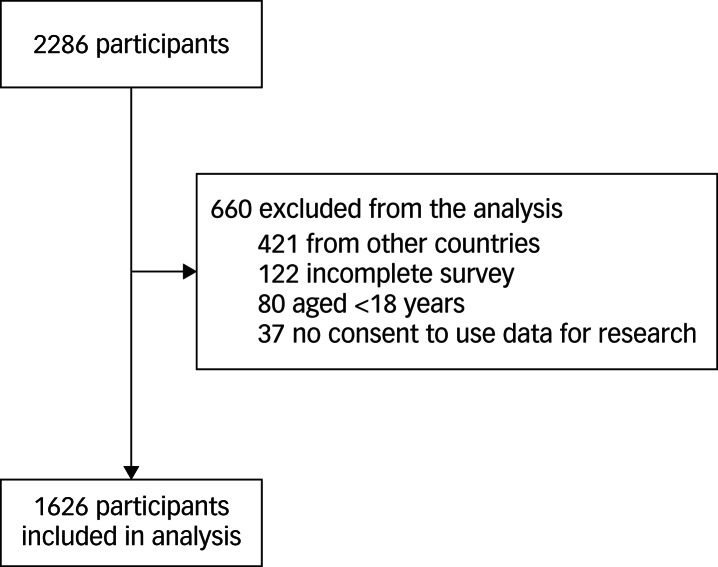
Study flow diagram.

A total of 2286 participants completed our online survey. We analysed data for 1626 (71.1%) of these participants, after application of predefined exclusion criteria (Fig. 1).

Table 1 presents the sociodemographic characteristics of the entire sample. Most participants were women (75.6%), with a mean age of 32.1 ± 10.5 years. The mean days in isolation were 10.6 ± 8.4 days (median 8 days) and half of the people (50.6%) were spending their isolation period with two to four people in their household. Most participants had no known contact (80.1%) or indirect contact (14.4%) with COVID-19. Most respondents were not under regular psychiatric care (90.9%), and only 170 participants had a previously known mental health condition (of which 158 had a depressive or anxiety disorder). As for the current work arrangements at the time of the survey, over half of the participants were working from home (56.7%), with just 16.1% working at their regular workplaces – which means the remainder of the respondents were either employed but not working, or unemployed. A total of 232 participants were taking at least one type of psychiatric medication: 8.8% (*n* = 143) were taking antidepressants, 7.4% (*n* = 120) were taking anxiolytics, 1.1% (*n* = 18) were taking mood stabilisers, 0.6% (*n* = 9) were taking stimulants and 0.4% (*n* = 7) were taking antipsychotics.

**Table 1:** Sociodemographic characteristics of the sample

	Participants (*N* = 1626)
Age, years, mean (s.d.)	32.1 (10.5)
Gender, % (*n*)	
Women	75.6 (1229)
Men	24.4 (397)
Occupation, % (*n*)	
Unemployed	3.8 (61)
Student	22.3 (363)
Healthcare professional	21.2 (345)
Other professions	51.2 (833)
Retired	1.5 (24)
Work arrangement, % (*n*)	
Working at workplace	16.1 (262)
Working from home	56.7 (922)
Not working	27.2 (442)
Days in isolation, mean (s.d.)	10.5 (8.4)
Number of other people in household, % (*n*)	
0	14.9 (242)
1	28.5 (463)
2–4	50.6 (823)
≥5	6.0 (98)
Known contact with COVID-19, % (*n*)	
None	80.1 (1303)
Indirect contact with COVID-19	14.4 (234)
Direct contact with COVID-19	3.1 (50)
Probably infected	2.0 (32)
Infected	0.4 (7)
Recovered	0.0 (0)
Psychiatric care, % (*n*)	9.1 (148)
Receiving psychiatric medication, % (*n*)	14.3 (232)

The mean depression score of the 1626 respondents was 7.4 ± 6.7, with BDI scores ranging from 0 to 47, and the mean anxiety score was 10.2 ± 8.2, with BAI scores ranging from 0 to 52. The mean insomnia score in the same sample was 6.4 ± 5.2, with ISI scores ranging from 0 to 26. Regarding the severity categories, we found that a substantial proportion of respondents had at least mild symptoms of depression (*n* = 491, 30.2%), anxiety (*n* = 864, 53.1%) and insomnia (*n* = 591, 36.3%). As for the moderate to severe categories, we found a smaller proportion of respondents with depression (*n* = 115, 7.1%), anxiety (*n* = 354, 21.8%) and insomnia (*n* = 136, 8.4%) symptoms. Interestingly, a significant proportion of participants who were not under psychiatric care presented some degree of clinical symptoms: 26.6% of the 1478 participants under no psychiatric care had at least mild depressive symptoms, 50.3% had at least mild anxiety symptoms and 33.5% had at least subthreshold insomnia symptoms.

Each of the sociodemographic and clinical variables collected in our survey was then used in univariate analyses as possible risk factors for depression, anxiety and insomnia, to determine if they could be included in the multivariate regression model. Univariate risk factors for the clinical outcomes are depicted in Table 2.

**Table 2:** Univariate analyses of risk factors for depression, anxiety and insomnia

Variables	Depression (BDI)		Anxiety (BAI)		Insomnia (ISI)	
	Univariate analysis		Univariate analysis		Univariate analysis	
	β (95% CI)	*P*-value	β (95% CI)	*P*-value	β (95% CI)	*P*-value
Age	−0.07 (−0.10 to −0.04)	<0.001	−0.04 (−0.08 to 0.00)	0.047	−0.02 (−0.04 to 0.00)	0.090
Male gender	−1.97 (−2.73 to −1.22)	<0.001	−4.02 (−4.92 to −3.11)	<0.001	−1.02 (−1.61 to −0.43)	0.001
Occupation						
Unemployed	3.44 (1.72−5.17)	<0.001	3.03 (0.91−5.15)	0.005	2.51 (1.16−3.86)	<0.001
Student	2.11 (1.29−2.93)	<0.001	1.24 (0.24−2.25)	0.015	0.78 (0.14−1.42)	0.017
Healthcare professional	−0.24 (−1.07 to 0.59)	0.577	−0.73 (−1.76 to 0.29)	0.160	−0.43 (−1.08 to 0.22)	0.199
Retired	0.51 (−2.18 to 3.20)	0.710	0.49 (−2.82 to 3.80)	0.771	0.40 (−1.71 to 2.51)	0.712
Other	Reference		Reference		Reference	
Work arrangement						
Workplace	Reference		Reference		Reference	
Remote working	0.12 (−0.80 to 1.04)	0.800	−0.22 (−1.34 to 0.90)	0.702	0.28 (−0.44 to 1.00)	0.443
Not working	1.45 (0.43−2.47)	0.005	1.13 (−0.12 to 2.38)	0.075	1.06 (0.26−1.85)	0.009
Days in isolation	0.09 (0.05−0.13)	<0.001	0.01 (−0.04 to 0.06)	0.699	0.05 (0.02−0.08)	0.001
Number of other people in household						
0	Reference		Reference		Reference	
1	−0.62 (−1.66 to 0.42)	0.244	0.16 (−1.12 to 1.43)	0.808	−0.47 (−1.28 to 0.34)	0.256
2–4	0.09 (−0.87 to 1.05)	0.860	0.44 (−0.73 to 1.62)	0.460	0.49 (−0.25 to 1.24)	0.195
≥5	−0.17 (−1.74 to 1.41)	0.834	0.41 (−1.51 to 2.33)	0.677	0.11 (−1.12 to 1.33)	0.862
Contact with COVID-19						
None	Reference		Reference		Reference	
Indirect	0.31 (−0.62 to 1.25)	0.509	0.55 (−0.58 to 1.69)	0.338	0.09 (−0.64 to 0.81)	0.814
Direct	−0.52 (−2.42 to 1.37)	0.587	−1.20 (−3.51 to 1.10)	0.306	−0.66 (−2.14 to 0.82)	0.381
Probably infected	3.03 (0.68−5.38)	0.011	4.95 (2.08−7.81)	0.001	1.60 (−0.23 to 3.43)	0.087
Infected	−2.20 (−7.18 to 2.77)	0.386	−3.37 (−9.44 to 2.69)	0.276	−2.68 (−6.57 to 1.20)	0.175
Recovered	–	–	–	–	–	–
Under psychiatric care	6.03 (4.93−7.13)	<0.001	8.24 (6.92−9.57)	<0.001	4.24 (3.38−5.10)	<0.001
Receiving psychiatric medication	4.73 (3.83−5.64)	<0.001	7.03 (5.95−9.12)	<0.001	3.56 (2.85−4.26)	<0.001

BDI, Beck Depression Inventory; BAI, Beck Anxiety Inventory; ISI, Insomnia Severity Index.

Of all the variables examined, univariate analyses found that older age and being male were protective for depressive symptoms with statistical significance; on the other hand, being unemployed, being a student, not currently working, higher number of days in isolation, the perceived notion of being infected without confirmation, being under psychiatric care and taking psychiatric medication were risk factors for depressive symptoms with statistical significance. As for anxiety symptoms, univariate analyses also found that older age and being male were protective, with statistical significance. Being unemployed, being a student, having the perception of being infected without confirmation, having psychiatric follow-up and taking psychiatric medication were significantly associated with higher anxiety symptoms. Regarding insomnia, univariate analyses showed that being male had a protective role, but no significant association was found with age (*P* = 0.090). Being unemployed, being a student, not currently working, higher number of days in isolation, being under psychiatric follow-up and taking psychiatric medication were significantly associated with more insomnia.

Multivariate regression analyses were then performed to identify independent risk factors for depression, anxiety and insomnia symptoms, with correction for potential confounders. Tables 3, 4 and 5 show the multivariate regression models for depression, anxiety and insomnia, respectively, with the statistically significant sociodemographic and clinical variables.

**Table 3:** Multivariate analysis of risk factors for depression

Variables	Multivariate analysis for depression	
	β (95% CI)	*P*-value
Age	−0.07 (−0.11 to −0.03)	<0.001
Male gender	−1.53 (−2.25 to −0.81)	<0.001
Occupation		
Unemployed	2.20 (0.51−3.89)	0.011
Student	0.96 (0.01−1.91)	0.048
Healthcare professional	−0.91 (−1.82 to −0.01)	0.047
Retired	0.63 (−2.13 to 3.39)	0.654
Other	Reference	
Work arrangement		
Workplace	Reference	
Remote working	−1.41 (−2.43 to −0.39)	0.007
Not working	0.12 (−0.93 to 1.16)	0.824
Days in isolation	0.06 (0.02−0.10)	0.004
Contact with COVID-19		
None	Reference	
Indirect	0.83 (−0.05 to 1.71)	0.066
Direct	−0.24 (−2.04 to 1.55)	0.789
Probably infected	3.32 (1.12−5.52)	0.003
Infected	−1.09 (−5.76 to 3.59)	0.648
Recovered	−	−
Under psychiatric care	3.63 (2.63−5.02)	<0.001
Receiving psychiatric medication	2.91 (1.76−4.06)	<0.001

**Table 4:** Multivariate analysis of risk factors for anxiety

Variables	Multivariate analysis for anxiety	
	β (95% CI)	*P*-value
Age	−0.04 (−0.08 to 0.00)	0.054
Male gender	−3.74 (−4.60 to −2.88)	<0.001
Occupation		
Unemployed	1.94 (−0.08 to 3.97)	0.060
Student	0.78 (−0.29 to 1.86)	0.154
Healthcare professional	−1.49 (−2.57 to −0.41)	0.007
Retired	0.38 (−2.91 to 3.67)	0.822
Other	Reference	
Work arrangement		
Workplace	Reference	
Remote working	−1.66 (−2.86 to −0.46)	0.007
Not working	−0.26 (−1.50 to 0.98)	0.684
Contact with COVID-19		
None	Reference	
Indirect	1.17 (0.12−2.23)	0.029
Direct	−0.98 (−3.13 to 1.17)	0.372
Probably infected	5.13 (2.50−7.76)	<0.001
Infected	−1.96 (−7.55 to 3.63)	0.492
Recovered	−	−
Under psychiatric care	4.35 (2.70−6.00)	<0.001
Receiving psychiatric medication	4.69 (3.32−6.07)	<0.001

**Table 5:** Multivariate analysis of risk factors for insomnia

Variables	Multivariate analysis for insomnia	
	β (95% CI)	*P*-value
Age	−0.03 (−0.05 to 0.00)	0.081
Male gender	−0.79 (−1.36 to −0.21)	0.007
Occupation		
Unemployed	1.81 (0.46−3.16)	0.009
Student	0.24 (−0.52 to 1.00)	0.538
Healthcare professional	−0.60 (−1.32 to 0.13)	0.106
Retired	−0.09 (−2.30 to 2.11)	0.935
Other	Reference	
Work arrangement		
Workplace	Reference	
Remote working	−0.55 (−1.37 to 0.27)	0.188
Not working	0.22 (−0.62 to 1.05)	0.610
Days in isolation	0.04 (0.01−0.07)	0.021
Contact with COVID-19		
None	Reference	
Indirect	0.43 (−0.27 to 1.14)	0.227
Direct	−0.52 (−1.95 to 0.92)	0.482
Probably infected	1.77 (0.01−3.52)	0.049
Infected	−1.83 (−5.57 to 1.91)	0.336
Recovered	−	−
Under psychiatric care	2.34 (1.24−3.45)	<0.001
Receiving psychiatric medication	2.31 (1.39−3.22)	<0.001

The multivariate regression model for depression showed that older age and being male retained statistical significance as protective variables for depression. Being a healthcare professional and working from home were also associated with lower depression symptoms. Being a student, being unemployed, higher number of days in isolation, the perception of infection without confirmation, being under out-patient psychiatric care and taking psychiatric medication were all variables significantly associated with more depressive symptoms. On the other hand, in contrast to the univariate analysis, the variable ‘not currently working’ was no longer statistically significant after adjusting for confounding factors.

In the multivariate regression model for anxiety, three variables were associated with lower anxiety symptoms: being male, being a healthcare professional and working remotely. Although there was also a trend for older age to have a protective effect on anxiety symptoms, this variable was not found to be statistically significant (*P* = 0.054). The perception of infection, being under psychiatric care and taking psychiatric medication were all statistically significant risk factors for anxiety, as observed by previous univariate models. Indirect contact with COVID-19 was also a significant factor. Being unemployed or a student, previously significant predictors in the univariate analysis, were no longer statistically significant in this model.

Regarding the model for insomnia, being male retained statistical significance as a protective variable for insomnia. In addition, older age showed a trend for being a protective factor, but it failed to demonstrate statistical significance (*P* = 0.081). Compared with the univariate models, being unemployed, higher number of days in isolation, being under out-patient psychiatric care and taking psychiatric medication remained statistically significant risk factors for insomnia. On the other hand, students were no longer a statistically significant risk factor for insomnia, and the perception of being infected gained significance.

## Discussion

The results of our study demonstrate that over a quarter of participants surveyed during the first wave of the COVID-19 pandemic showed depression scores compatible with at least mild depression symptoms, over half presented with scores of at least mild anxiety symptoms and over a third had scores compatible with at least subthreshold insomnia symptoms. Although we used different methodology, these scores were higher than the ones observed in epidemiological samples of the Portuguese pre-pandemic population, which showed that 9.32% had depression, 6.06% had anxiety disorders^16^ and 27.7% had insomnia symptoms.^17^

In another study assessing the Portuguese population during the first wave, 49.2% of the respondents reported a moderate to severe psychological effects of the COVID-19 pandemic.^24^ However, when compared with our study, a lower proportion of individuals reporting at least mild depression (20.1 *v*. 30.2%) and anxiety symptoms (27.2 *v*. 53.1%) was found. This difference might be secondary to the use of different rating scales or the different time frames used (the first month of the state of emergency versus a 3-day period a week after the state of emergency started). Nonetheless, both studies report higher levels of anxiety and depression symptoms when compared with the pre-pandemic Portuguese population samples. Although causality could not be established, the higher severity of symptoms was potentially secondary to psychological effects associated with the COVID-19 pandemic. Nevertheless, it is important to highlight that these results may indicate a transitory increase in depressive, anxious and insomnia symptoms, which will not necessarily progress to the development of a disorder. According to a longitudinal study by Fancourt et al,^25^ conducted during the first lockdown in England, people started to recover as time went by, presenting with lower levels of symptoms, suggestive of an adaptive process to their circumstances.

Furthermore, we showed that female gender, being under psychiatric care, taking psychiatric medication, probable infection and being unemployed (except for anxiety) was associated with higher levels of depressive, anxiety and insomnia symptoms. Another study evaluating a Portuguese population during the pandemic's first wave (*n* = 1280) reported that being male, older age, active working, having a garden and practicing physical exercise were protective factors for mental health symptoms, which is in line with our findings.^26^ Scores of depression and insomnia were also higher in those isolating for more days, with an average increase of 0.597 points in BDI and 0.381 in ISI for every extra 10 days of physical distancing, which suggests that in the first stage of lockdown, the duration of isolation may have had a direct effect on depression and insomnia symptoms. Surprisingly, the same was not found for anxiety symptoms. It was also observed that people working from home presented lower depression and anxiety scores than those working at their workplace or not currently working, suggesting that working with a lower infection risk might attenuate eventual psychological effects during this period.

Interestingly, and similar to other studies,^25,27^ younger participants presented with significantly higher depressive scores in our cohort (*P* < 0.001), falling short of statistical significance for both anxiety and insomnia (*P* = 0.054 and *P* = 0.081, respectively). Every additional 10 years of age resulted in an average decrease of 0.697 points in the BDI score. The student category remained statistically significant for depressive symptoms, even after adjusting for confounding factors such as age in the multivariate regression model. This highlights that being a student on its own is a significant risk factor for depressive symptoms during the isolation period. These findings may be explained by the fact that young people tend to receive information from anxiety-provoking social media, allied to the fact that, in Portugal, universities interrupted classroom teaching before most other activities halted, with many students likely having to return to their parents’ homes, and as a consequence, losing some level of independence and preventing them from the usual social interaction with peers. Curiously, this intriguing association between student status and depressive symptoms had already been reported in China by Wang et al,^15^ reflecting probable commonalities in the way isolation is experienced in different cultures. Absence of short-/medium-term goals – an issue that students share with the unemployed, who also present higher depression scores – might also be contributing to this issue.

Regarding psychiatric medication, it was interesting that there was a gap between individuals with previous psychiatric conditions (*n* = 170) and respondents under psychiatric medication (*n* = 232). We hypothesise that this could be an indicator of early use of anxiolytics (*n* = 120) without formal medical guidance.

Contrary to the results found in recent studies focused on mental health outcomes of healthcare professionals,^28–30^ our multivariate regression models showed that healthcare professionals who participated in our study had lower depression and anxiety scores than the rest of the professional groups. As noted by recent reviews on the impact of COVID-19^30^ and previous virus outbreaks^31^ on the mental health of healthcare workers, there are several mitigating factors that might explain to a certain degree these results. At the time data collection took place, the pandemic was at an early stage with relatively swift state intervention, implementing distancing measures that allowed the National Health System to cope with the influx of patients.^32^ As such, the workload was not as heavy and there was not a need for a generalised front-line intervention from healthcare professionals.

Participants with indirect contact with COVID-19 in our cohort presented with higher anxiety levels. This result highlights the importance of testing those with COVID-19 symptoms in the general population, not only for public health reasons, but also because of the potential effects of the absence of a formal diagnosis. Similar results were found in the UK,^27^ where having a confirmed or suspected infection of COVID-19 were associated with screening positive for general anxiety disorder and depression.

Contrary to the findings reported from other studies,^25,33^ our results showed that living alone was not associated with higher levels of depression, anxiety or insomnia symptoms. We hypothesise that this could be explained by the characteristics of the current digital age. Through the power of social media and technology, being in confinement alone was not synonymous with social isolation.^34^ People were still able to stay virtually connected, even if keeping their physical distance, which might explain to some extent our findings. In fact, previous studies^35,36^ have shown that being alone was not synonymous with feeling lonely, and that the company of others does not necessarily prevent loneliness. As such, feeling lonely, which we did not formally investigate, might be a better predictor for adverse mental health symptoms during this period of physical isolation.^37^

### Strengths and limitations

There are several limitations in this study. First, since this study's design was cross-sectional, we could not make causal inferences. Longitudinal studies would be ideal to understand how the time spent in physical distancing modulates mental health. Second, the sample is not representative of the Portuguese population. It had a higher percentage of women and a younger age distribution when compared with the average Portuguese population (www.ine.pt; accessed on 30 March 2021). However, the methods used allowed for a quick questionnaire implementation and simple enrolment of participants. Third, the BDI and BAI scales are known to overestimate the levels of symptoms,^38,39^ which may influence the depressive and anxiety results. Nevertheless, we considered using these scales as they were both validated for the Portuguese population,^21,22^ and are still a mainstay in the rapid screening and assessment of mental health.^40^

On the other hand, we believe this study has some strengths by using a questionnaire that is not only broad in the mental health dimensions evaluated (depression, anxiety and insomnia), but also provides adequate coverage of the most likely predictors of such dimensions.

In conclusion, COVID-19-related distancing measures seem to be associated with high levels of adverse mental health symptoms. In this study, several risk factors were shown to be associated with higher levels of depression, anxiety and insomnia symptoms. This highlights the importance of paying particular attention to mental health during physical distancing periods, particularly when a result of highly infectious diseases, such as the COVID-19 pandemic.

Particular attention and support should be given to vulnerable groups, such as women, younger adults, psychiatric patients, students and the unemployed, through the local health system, employers, education institutions and employment centres.

## Data Availability

The data that support the findings of this study are available from the corresponding author, H.P.R., upon reasonable request. The data are not publicly available because they contain sensitive data that might compromise the privacy of the research participants.
